# Immunoglobulin G galactosylation and sialylation are associated with pregnancy-induced improvement of rheumatoid arthritis and the postpartum flare: results from a large prospective cohort study

**DOI:** 10.1186/ar2892

**Published:** 2009-12-16

**Authors:** Fleur E van de Geijn, Manfred Wuhrer, Maurice HJ Selman, Sten P Willemsen, Yaël A de Man, André M Deelder, Johanna MW Hazes, Radboud JEM Dolhain

**Affiliations:** 1 Department of Rheumatology, Erasmus MC University Medical Center Rotterdam, Dr. Molewaterplein 50, 3015 GE Rotterdam, The Netherlands; 2 Biomolecular Mass Spectrometry Unit, Department of Parasitology, Leiden University Medical Center, Albinusdreef 2, PB 9503, Leiden, The Netherlands; 3 Department of Biostatistics, Erasmus MC University Medical Center Rotterdam, Dr. Molewaterplein 50, 3015 GE Rotterdam, The Netherlands

## Abstract

**Introduction:**

Improvement of rheumatoid arthritis (RA) during pregnancy has been causatively associated with increased galactosylation of immunoglobulin G (IgG) N-glycans. Since previous studies were small, did not include the postpartum flare and did not study sialylation, these issues were addressed in the present study.

**Methods:**

Serum from 148 RA cases and 32 healthy controls was collected at several time points before, during and after pregnancy. Improvement during pregnancy and postpartum flare were determined according to the European League Against Rheumatism (EULAR) response criteria. Galactosylation and sialylation of Immunoglobulin G (IgG) and the presence of bisecting N-acetylglucosamine (GlcNAc) were analyzed by matrix-assisted laser desorption/ionization - time of flight - mass spectrometry (MALDI-TOF-MS).

**Results:**

IgG1 and IgG2 galactosylation of the cases and controls increased during pregnancy with a maximum in the third trimester. Galactosylation decreased directly postpartum. IgG galactosylation of controls was at a higher level than cases (*P *< 0.001 at all time points) and a similar pattern was observed for sialylation. Moreover, there was a good association between galactosylation and sialylation. The increase in galactosylation was significantly more pronounced for cases with improvement than cases without improvement during pregnancy. The reverse was true for deteriorators and non-deteriorators postpartum. The presence of bisecting GlcNAc was not significantly influenced by pregnancy or postpartum for cases and controls.

**Conclusions:**

This large cohort study demonstrates the association of changes in galactosylation with both pregnancy-induced improvement and postpartum flare in RA-patients, suggesting a role for changes in glycosylation in the pregnancy-induced improvement of RA.

## Introduction

Pregnancy is the only natural situation that results in spontaneous improvement of rheumatoid arthritis (RA) and a flare after delivery. Insight into this mechanism may not only enlarge our knowledge on pregnancy-induced improvement of RA, but may also contribute to a better understanding of pathogenic factors involved in RA in general. It has been suggested that pregnancy-related changes in the glycosylation of immunoglobulins might mediate these changes in disease severity [1,2].

**Figure 1:**
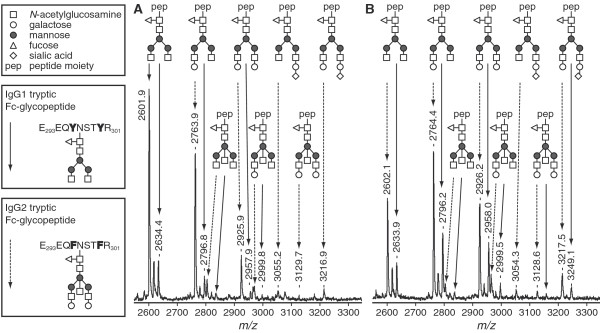
MALDI-TOF-MS analysis of tryptic glycopeptides of IgG1 and IgG2. A representative sample of an RA-patient before pregnancy **(a) **and in the third trimester **(b) **is shown. Glycopeptides derived from IgG1 and IgG2 were analyzed for galactosylation and sialylation in the reflectron positive mode. Glycopeptides of IgG 1 are indicated by continued arrows, while glycopeptides of IgG2 are indicated by striated arrows. Three glycoforms of IgG1 have been found to be below the detection limit of the MALDI-TOF-MS method in this sample as well as in several other samples.

For immunoglobulin G (IgG) multiple glycoforms can be identified due to the presence of a single N-glycan chain attached to each IgG fragment crystallizable (Fc) portion [3]. This N-glycan shows heterogeneity due to the presence or absence of fucose, galactose or sialic acid residues and bisecting N-acetylglucosamine (GlcNAc) (Figure 1) [4,5]. Regarding galactosylation, three subfamilies called either galactose-0 (Gal-0) (agalactosyl IgG, no galactose), Gal-1 (galactose on one arm) or Gal-2 (galactoses on both arms) have been defined [6]. On the Gal-1 and Gal-2 glycans one terminal sialic acid residue can be present.

In RA-patients higher levels of agalactosyl IgG are found compared to controls and this is associated with increased disease activity and more disease progression [4,7]. Moreover, in two small studies increased galactosylation during pregnancy has been associated with the pregnancy-induced improvement of RA [1,2]. Due to the small sample size and limited follow-up period these studies could not provide detailed description of the changes in galactosylation during pregnancy and postpartum. Fucosylation seems not to be related to RA or pregnancy [8], whereas sialylation and the presence of the *bisecting *GlcNAc have not yet been studied in these settings. Moreover these studies applied the lectin analysis method or the GN7 antibody ELISA to detect the galactosylation level, however, both of which could not analyze the Fragment crystalizable (Fc) and Fragment antigen-binding (Fab) glycosylation separately and its accuracy was questioned. Now, the MALDI-TOF-MS method which is now applied can investigate the Fc fragment galactosylation and the position of bisecting GlcNAc with great accuracy and reproducibility.

The aim of the present study is to investigate the changes in IgG glycosylation in detail (galactosylation, sialylation and the presence of the *bisecting *GlcNAc) in a large cohort of 148 RA-patients and 32 controls from pre-pregnancy onwards until six months postpartum, together with associations with disease activity and medication use as well as other factors.

## Materials and methods

### Study population

The present study is embedded in the PARA-study (Pregnancy-induced Amelioration of Rheumatoid Arthritis), a prospective cohort study on pregnancy and RA [9]. Data of the first 148 Caucasian RA-patients (cases) are included. Thirty-two healthy pregnant Caucasian volunteers without adverse obstetric history served as controls. All participants gave informed consent. The study is in compliance with the Helsinki Declaration and approved by the Ethics Review Board at the Erasmus MC University Medical Center Rotterdam.

### Data collection

N = 57 cases were followed from pre-pregnancy, n = 65 cases from the first trimester, n = 14 cases from the second trimester and n = 12 cases from the third trimester and onwards, all controls from first trimester and onwards.

Disease activity was scored using a disease activity score (DAS28) with three variables (swollen joint count, tender joint count and a C-reactive protein (CRP) level) [10,11].

### Categorization of disease activity and clinical response

According to the EULAR criteria, remission of RA was defined as DAS28<2.6 and intermediate and high disease activity as DAS28>3.2 [12]. Improvement during pregnancy was defined according to the EULAR criteria as *good, moderate *(combined to *responders*) or *non*-responders [12]. The response criteria can only be applied to those patients with an initial DAS28>3.2 at first trimester (n = 75). A postpartum flare was defined according to so called *reversed *EULAR criteria [9,12]. Since there is no baseline DAS28 requirement for these criteria, this classification was applied to all cases. An early flare was defined when deterioration began between six weeks and three months postpartum, a late flare with deterioration between three to six months postpartum.

### IgG glycosylation analysis

IgG was purified from sera using Protein A-Sepharose beads (GE Healthcare, Eindhoven, The Netherlands) followed by trypsinisation as described previously with minor alterations [13]. These beads bind IgG1, IgG2 and IgG4, but not IgG3. Then the resulting glycopeptides were purified by reverse phase- solid phase extraction (SPE) (Supelco DSC-18 plate SPE-96 (Sigma, Zwijndrecht, The Netherlands)) and eluted into a V-bottom 96-well microtitration plate (Nunc, Roskilde, Denmark) using 200 μl 18% acetonitrile (AcN) containing 0.1% trifluoroacetic (TFA). Glycopeptide samples were dried by vacuum centrifugation and dissolved in water.

Galactosylation of IgG1 and IgG2 and the incidence of bisecting GlcNAc were analyzed for all samples: aliquots of the glycopeptide samples after reverse phase purification were spotted on a polished steel 384-positions MALDI-TOF-MS target plate and allowed to dry. Sample spots were overlaid with α-cyanocinnamic acid matrix (5 mg/ml in 50% AcN) and allowed to dry, resulting in a microcristalline sample preparation. Glycopeptides were analyzed in the reflectron positive mode on an Ultraflex II MALDI-TOF-MS (Bruker Daltonics, Bremen, Germany). N = 100 shots were acquired per position, and spectra were acquired from n = 30 different positions per spot, resulting in a sumspectrum obtained by accumulation from 3,000 spectra per sample spot.

For the analysis of sialylation, aliquots of the glycopeptide samples were spotted on a mitrotiter plate (MTP) AnchorChip 600/384 plate (Bruker Daltonics) and allowed to dry. Sample spots were overlaid with 2,5-dihydroxybenzoic acid matrix (5 mg/ml in 50% AcN with 0.1% TFA) and allowed to dry, resulting in a macrocristalline sample preparation. Glycopeptides were analyzed by MALDI-TOF-MS in the linear positive mode. Per sample spot 2,000 spectra were accumulated. These analyses were performed in three subgroups: first, n = 10 cases and n = 10 controls randomly selected from our cohort at every timepoint before (cases), during and after pregnancy. Second, sialylation was determined in n = 15 responders and n = 15 non-responders selected upon the most pronounced and the least-pronounced changes in disease activity during pregnancy. Third, sialylation was determined in n = 15 cases with a flare early postpartum and n = 15 cases without a flare selected upon the most pronounced and the least-pronounced changes in disease activity postpartum.

Mass spectra were processed in FlexAnalysis (Bruker Daltonics) with baseline subtraction and peak detection of the IgG1 and IgG2 glycopeptide signals. Peak lists were imported into Excel.

To determine the inter- and intra-day variation on every plate one or more standard sera were added and measured.

From the MALDI-TOF-MS measurements in the reflectron positive mode, IgG1 and IgG2 signals for six glycoforms were analyzed: Gal-0 without bisecting GlcNAc, Gal-1 without bisecting GlcNAc, Gal-2 without bisecting GlcNAc and Gal-0+bisecting GlcNAc (G0+N), Gal-1+bisecting GlcNAc (G1+N), Gal-2+bisecting GlcNAc (G2+N).

All analyzed glycopeptides contain fucose residues. Due to relative low incidence (approximately 5%) and overlap with IgG4 glycoforms, the applied analytical approach did not allow the analysis of non-fucosylated glycopeptides.

The levels of galactosylation of IgG1 and IgG2 without bisecting GlcNAc were calculated on the basis of signal heights observed in MALDI-TOF-MS. Based upon these signal heights a percentage of galactosylation was determined. This percentage represents the actual number of galactoses present on the outer arms of the N-glycan chain (1 Gal on Gal-1 and 2 Gal on Gal-2) divided by the total number of available antenna positions for galactosylation on the outer arms of the N-glycan (two available antenna positions both on Gal-0, Gal-1 as well as on Gal-2). It was calculated using the following term:

The incidence of bisecting GlcNAc on IgG1 and IgG2 was also calculated on the basis of the signal heights observed in MALDI-TOF-MS. On the basis of these signal heights a percentage of the presence of GlcNAc (N) was determined. This percentage represents all signal heights with GlcNAc present on the N-glycan with or without galactoses (Gal-0+N, Gal-1+N or Gal-2+N) divided by all available signal heights with and without GlcNAc using the following term:

Based on the MALDI-TOF-MS measurements in the linear positive mode, the incidence of sialic acid (SA) per galactose moiety on IgG1 and IgG2 was calculated on the basis of signal heights observed in MALDI-TOF-MS. Based upon these signal heights a percentage of sialylation was determined. This percentage represents the actual number of sialic acid sugar moieties present on the galactose sugar moieties on outer arms of the N-glycan chain (one SA on Gal-1 and one or two SA on Gal-2) divided by the total number of available antenna positions for sialic acid on the galactose sugar moieties on the outer arms of the N-glycan (two available positions on Gal-2 with or without SA and one available position on Gal-1 with or without SA). It was calculated using the following term:

### Statistical analysis

Statistical analysis was performed using the Statistical Package for the Social Sciences (SPSS) 15.0 and Statistical Analysis Software (SAS) 9.1. A two-sided *P*-value ≤ 0.05 was considered statistically significant.

A Linear Mixed Model (LMM) with unstructured residual correlation was used to test for differences in the galactosylation and sialylation at each timepoint and for changes in time between cases and controls as well as responders versus non-responders and flare versus no-flare postpartum. Pearson and Spearman rank tests were used to determine possible associations.

A multivariate analysis, conditional on the timepoint of visit, was performed to investigate which covariates determine the level of galactosylation. A constant effect in time was assumed. Only covariates with a *P*- value < 0.20 in the univariate analysis were introduced in the multivariate analysis. The following covariates were tested: use of salazopyrine, prednisone, methotrexate or biologicals, DAS28, presence of joint erosions, rheumatoid factor (RF) positivity and anti-cyclic citrullinated peptide (anti-CCP) positivity, breast feeding and maternal age at delivery.

Finally, to determine whether changes in galactosylation precede changes in disease activity, for every timepoint interval the change in IgG galactosylation was divided by the total change in galactosylation. The change in disease activity was also calculated per interval and divided by the total change in disease activity. This resulted in a percentage of change per timepoint interval. A paired sample *t*-test tested for equality.

## Results

### Description of study cohort

All cases (n = 148) fulfilled the American College of Rheumatology (ACR) 1987 revised criteria for RA (Table 1). Medication use of this cohort was described before [14]. In more detail: the use of sulfasalazine and prednisone through pregnancy and postpartum was documented as below. For sulfasalazine use 23 of 57 cases (40.4%) at pre-pregnancy, 37/118 (31.4%) in first trimester, 42/133 (31.6%) in second trimester, 43/146 (29.5%) in third trimester, 43/144 (29.9%) six weeks postpartum, 48/144 (33.3%) three months postpartum, 49/142 (34.5%) six months postpartum.

**Table 1:** Cohort characteristics

	Cases (n = 148)	Controls (n = 32)
Mean age at delivery in years (SD)	32.3 (3.8)	32.0 (4.4)
Median disease duration at delivery in years (range)	8.0 (0.7 to 29.7)	-
Number of nulliparous women, n (%)	70/147 (47.6)	14/32 (43.8%)
Mean gestational age at delivery, weeks (range)	39.3 (31.4 to 42.1)	40.4 (34.0 to 42.0)
Breastfeeding (six weeks postpartum), n (%)	62/148 (41.9)	27/32 (84.4)
Anti-CCP positive, n (%)	93/147 (63.3)	-
Rheumatoid Factor (IgM) positive, n (%)	108/148 (73.0)	-
Erosive disease, n (%)	43/147 (70.7)	
DAS28-CRP3 >3.2 in first trimester, n (%)	75 (61.5)	-
Classification of disease activity during pregnancy		
good response/moderate response	37/75 (49.3)	-
no response	38/75 (50.7)	-
Classification of disease activity during postpartum period (*early flare*)		
severe deterioration/moderate deterioration (n, %)	35/141* (24.8)	-
no deterioration (n, %)	106/141* (75.2)	-
Classification of disease activity during postpartum period (*late flare*)		
severe deterioration/moderate deterioration (n, %)	29/141* (20.6)	-
no deterioration (n, %)	112/141* (79.4)	-
Median number of DMARDs (incl prednisone) prior to conceive (min-max)	2 (0-7)	-
No DMARD** use prior to conceive, n (%)	7/147 (4.8)	
Use of methotrexate prior to conceive, n (%)	75/147 (51.0)	-

*n = 7 cases are missing, since a small proportion of DAS scores are missing.

** DMARDs: disease modifying anti-rheumatic drugs.

For prednisone use 19 of 57 cases (33.3%) at pre-pregnancy, 43/118 (36.4%) in first trimester, 48/133 (36.1%) in second trimester, 50/146 (34.2%) in third trimester, 49/144 (34.0%) six weeks postpartum, 48/144 (33.3%) three months postpartum, 46/142 (32.4%) six months postpartum.

The use of methotrexate and biologicals postpartum was documented as below: For methotrexate use postpartum 24 of 144 cases (16.7%) at six weeks postpartum, 40/144 (27.8%) three months postpartum, 56/142 (39.4%) six months postpartum.

For use of biologicals postpartum 7 of 144 cases (4.9%) at six weeks postpartum, 13/144 (9.0%) three months postpartum, 14/142 (9.9%) six months postpartum. Other DMARDs were only used by a very limited number of participants in this cohort both during (≤ 2.3%) and after pregnancy (≤ 7.6%).

### MALDI-TOF-MS measurements accuracy and reproducibility

The intraday and interday variability for the analyzed glycopeptides of IgG1 and IgG2 was below 4% and 6%, respectively. The IgG4 glycopeptides were not analyzed due to their low abundance.

### Galactosylation profiles during pregnancy and postpartum of cases and controls

**Figure 2:**
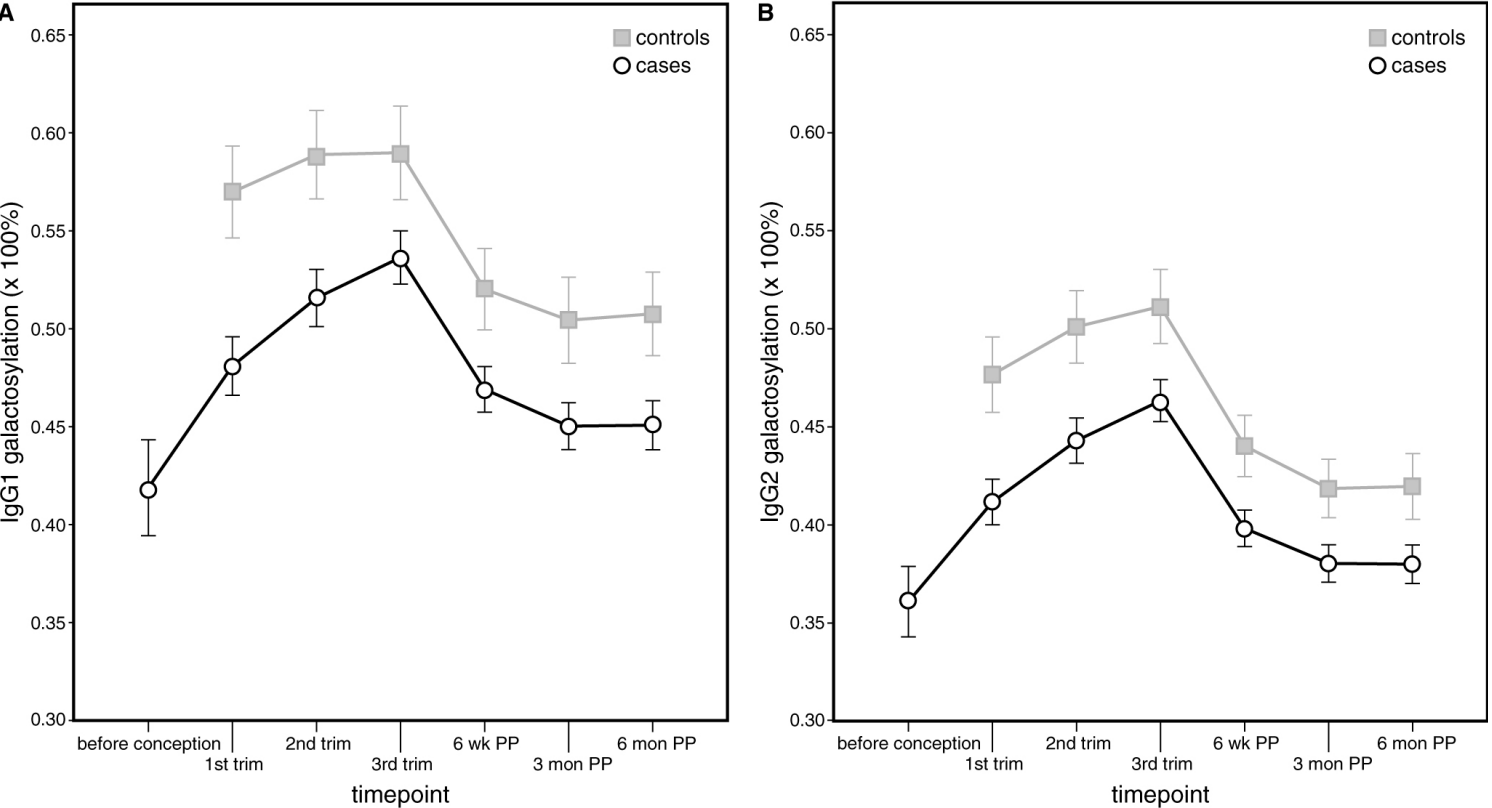
Mean galactosylation of IgG1 and IgG2 in cases and controls during pregnancy and postpartum. IgG1 **(a) **and IgG2 **(b) **galactosylation levels (in percentages) increase during pregnancy and decline postpartum. IgG1 and IgG2 galactosylation profiles of controls are at a significantly higher level than cases (*P *< 0.001, Linear Mixed Model at all timepoints). The vertical bars illustrate the 95% confidence intervals. Abbreviations: trim = trimester of pregnancy; wk = weeks; PP = postpartum; mon = months.

For cases an increase in IgG1 galactosylation was observed during pregnancy from preconception (mean 43.4% (standard deviation (SD) 8.3%) to first trimester (mean 48.4% (SD 8.4%) until the third trimester (53.7%, (SD 8.3%), *P *< 0.0001). After pregnancy a significant decrease in galactosylation was observed with lowest levels at six months postpartum (44.9% (SD 7.7%), *P *< 0.0001, Figure 2a). IgG2 galactosylation profiles show a similar pattern as IgG1 (Figure 2b). In the controls, IgG1 and IgG2 galactosylation profiles were at a significantly higher level than in cases (*P *< 0.001), and changes were less pronounced than in the cases (Figure 2).

### Galactosylation and disease activity levels

**Figure 3:**
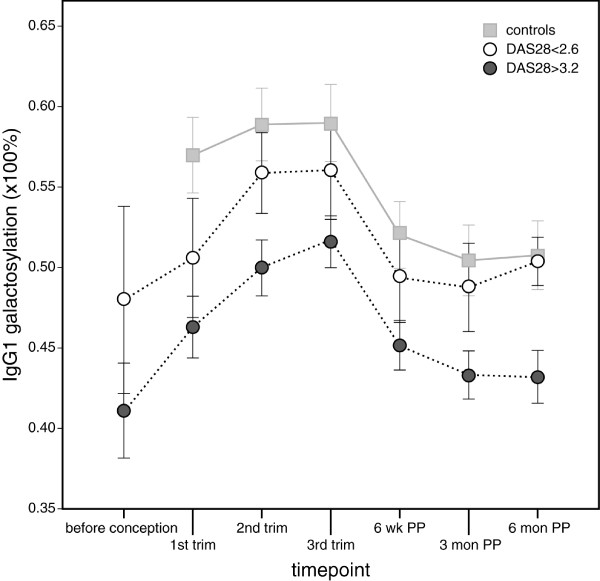
Mean IgG1 galactosylation levels in relation to rheumatoid arthritis disease activity levels. For this purpose at every timepoint all cases were divided in two categories; that is, those with a DAS28>3.2 or DAS28<2.6. Please note that each timepoint may include different RA-cases. For comparison controls are added to the graph. The IgG1 galactosylation level which is associated with disease remission (DAS28 <2.6) is dependent on the timepoint of measurement. Similar data were observed for IgG2 (data not shown). The vertical bars illustrate the 95% confidence intervals. Abbreviations: DAS28 = disease activity score; trim = trimester of pregnancy; wk = weeks; PP = postpartum; mon = months.

IgG1 and IgG2 galactosylation levels are associated with disease activity at every timepoint (Pearson correlation 0.35<*R*<0.49, *P *< 0.005). Lower disease activity levels show higher galactosylation levels, and resemble more the levels of the controls. Both IgG1 (Figure 3) and IgG2 (data not shown) galactosylation levels which are associated with disease remission (DAS28<2.6) depend on the timepoint of measurement.

### Changes in IgG galactosylation are associated with improvement of disease activity in responders and non-responders

**Figure 4:**
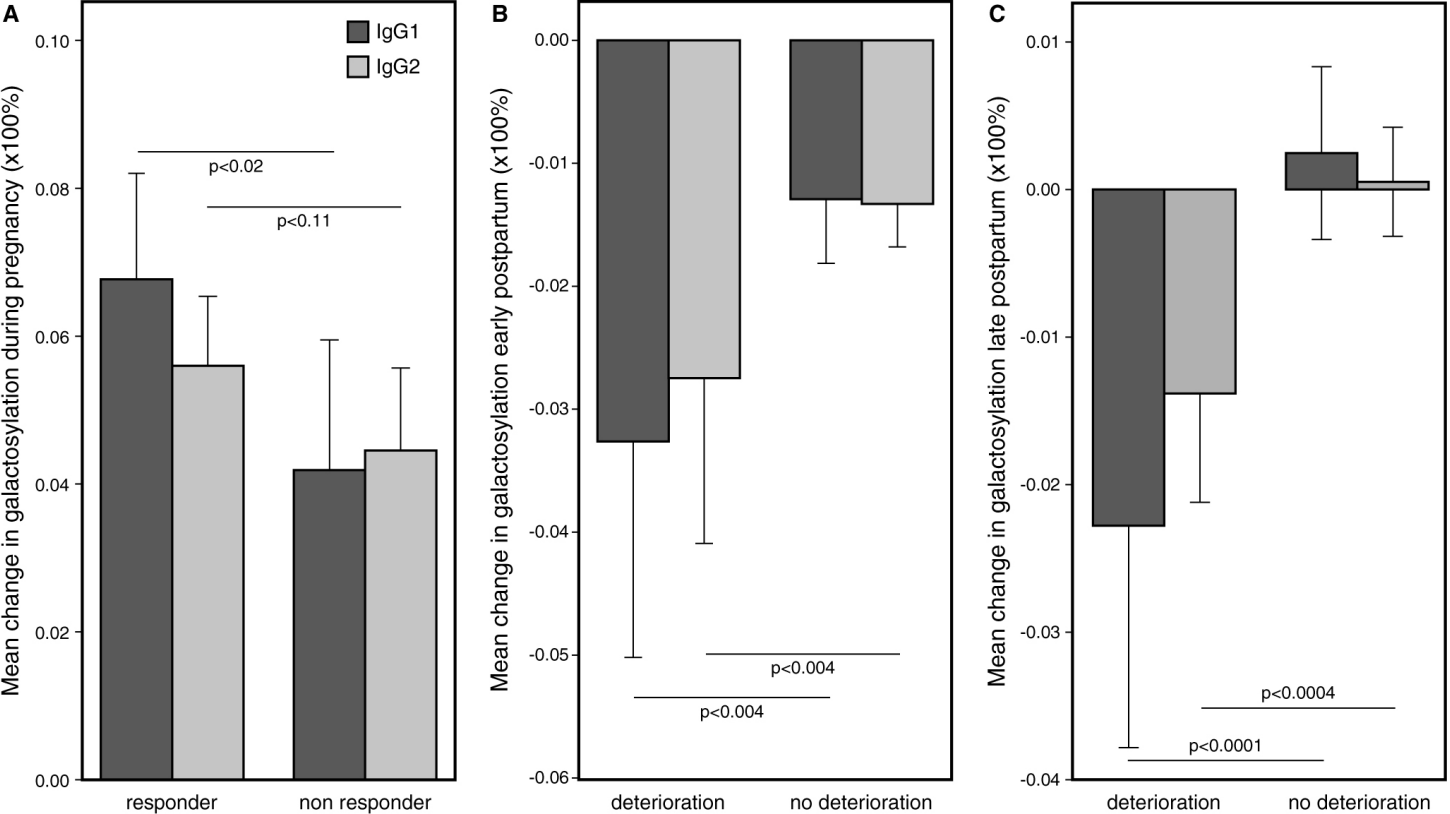
Mean change in IgG1 and IgG2 galactosylation during pregnancy and early or late postpartum. **(a) **Mean change in IgG1 and IgG2 galactosylation (×100%) during pregnancy in (good and moderate) responders according to the EULAR response criteria (cases that improved during pregnancy, n = 37) and non-responders (cases that did not improve during pregnancy, n = 38). The change in IgG galactosylation was significantly different between responders and non-responders for IgG1 (*P *< 0.02), whereas for IgG2 a trend towards significance could be observed (*P *= 0.11). **(b) **Mean change in IgG1 and IgG2 galactosylation (×100%) in the postpartum period in cases with an early flare between six weeks and three months postpartum (deterioration, n = 35) and cases without an early flare (no deterioration, n = 106). The change in galactosylation was significantly different between early flare and no early flare for IgG1 and IgG2 (*P *< 0.004). **(c) **Mean change in IgG1 and IgG2 galactosylation (×100%) in the postpartum period in cases with a late flare from three to six months postpartum (deterioration, n = 29) and cases without a late flare (no deterioration, n = 112). The change in galactosylation was significantly different between late flare and no late flare for IgG1 and IgG2 (*P *< 0.0001 and *P *< 0.0004, respectively). The vertical bars illustrate the 95% confidence intervals.

The change in galactosylation from the first to the third trimester was significantly different between responders (n = 37) and non-responders (n = 38) for IgG1 (6.8% (SD 0.80%) versus 4.2% (SD 0.79%), respectively, *P *< 0.02), whereas for IgG2 a trend could be observed (5.6% (SD 0.51%) versus 4.5% (SD 0.50%), respectively, *P *< 0.11, Figure 4a).

### Changes in IgG galactosylation are associated with flare postpartum

Cases with a late flare may also have experienced an early flare (n = 9). The change in galactosylation from six weeks to three months postpartum was significantly different between the cases with an early flare (n = 35) and without flare (n = 106) (-3.3% (SD 0.58%) versus -1.3% (SD 0.33%), respectively, for IgG1; -2.7% (SD 0.42%) versus -1.3% (SD 0.24%), respectively, for IgG2, both *P *< 0.004, Figure 4b). The change in galactosylation between three and six months postpartum was also significantly different between the cases with a late flare (n = 29) and without late flare (n = 112) (-1.2% (SD 0.49%) versus +0.58% (SD 0.33%), respectively, for IgG1, *P *< 0.0001 and -1.1% (SD 0.30%) versus +0.16% (SD 0.20%), respectively, for IgG2, *P *< 0.0004, Figure 4c).

### Galactosylation changes do not precede disease activity changes

When changes in galactosylation and changes in disease activity were tested for equality, this could not be rejected, indicating that galactosylation and DAS28 may change synchronically in time.

### IgG sialylation during pregnancy and postpartum

IgG sialylation was, like galactosylation, determined as total percentage of sialic acid (SA) residues per N-glycan. The presence of SA on IgG1 and IgG2 is low in the serum for cases and controls (all measurements taken together: mean 5.8%, SD 2.3% SA per N-glycan for IgG1 for cases (controls 7.1%, SD 2.7%) and 6.6%, SD 2.6% per N-glycan for IgG2 for cases (controls 7.9%, SD 2.5%)). In RA-cases N-glycan sialylation levels and IgG galactosylation were significantly correlated (Spearman rho 0.57 and 0.69 for IgG1 and IgG2, respectively, both *P *= 0.0001). In controls, the correlation between sialylation and IgG galactosylation was 0.77 and 0.72 for IgG1 and IgG2, respectively, both *P *= 0.0001).

The mean sialylation levels per N-glycan for IgG1 and IgG2 increased during pregnancy and decreased postpartum for cases (n = 10) and controls (n = 10). In cases, for IgG1, an increase in sialylation was observed during pregnancy from preconception (mean 5.01% (SD 0.83%) to first trimester (mean 6.47% (SD 0.69%) until second trimester (7.45% (SD 0.75%), *P *< 0.049). Third trimester: 7.27% (SD 0.82%). After pregnancy a decrease in sialylation was observed with lowest levels at six months postpartum (4.61% (SD 0.47%), *P *< 0.056). For IgG2, an increase in sialylation was observed during pregnancy from preconception (mean 5.45% (SD 0.11%) to first trimester (mean 6.59% (SD 0.64%) until second trimester (8.81% (SD 0.75%), *P *< 0.022). Third trimester: 8.52% (SD 0.77%). After pregnancy a decrease in sialylation was observed with lowest levels at six months postpartum (5.90% (SD 0.44%), *P *< 0.550). The controls showed a higher level of sialylation than the cases, but this difference was not significantly different (*P *< 0.280). The increase in N-glycan sialylation was larger in responders than in non-responders during pregnancy (for IgG1: within responders +1.8%, SD 0.42%, *P *= 0.0007; within non-responders +0.34%, SD 0.42%, *p *< 0.216; for IgG2: within responders +1.8%, SD 0.50%, *P *= 0.0008; within non-responders +1.0%, SD 0.42%, *P *< 0.052).

In the postpartum period no significant changes were observed for IgG1 sialylation between cases with or without flare. The change in IgG1 sialylation was for cases with early flare: +0.03% (SD 0.64%), *P *< 0.957, and for cases without early flare: -0.20% (SD 0.56%), *P *< 0.728%; for cases with a late flare: -0.81% (SD 0.73%), *P *< 0.271, and for cases without late flare -0.49% (SD 0.67%), *P *< 0.471.

For IgG2 sialylation a decrease in N-glycan sialylation could be observed in cases with an early flare (-0.95% (SD 0.40%), *P *= 0.024) and cases without an early flare (-.68%, (SD 0.40%), *P *< 0.095). For the late postpartum flare in IgG2 sialylation non-significant results were seen: -0.01% (SD 0.92%), *P *< 0.99, and for cases without late flare -0.02% (SD 0.92%), *P *< 0.98.

### Dependent variables of galactosylation

To investigate which factors determine the level of galactosylation multivariate analyses were performed. In the multivariate analyses only DAS28 and sulfasalazine (*P *= 0.06) for IgG1 and DAS28 and prednisone use for IgG2 had a significant negative effect on galactosylation.

### Presence of bisecting GlcNAc and its relation to galactosylation

The presence of IgG with a bisecting GlcNAc is low in the serum (first trimester mean 13.7%, SD 2.8% for IgG1 (both cases and controls) and mean 13.3%, SD 3.2% or 13.5%, SD 3.5% (cases and controls, respectively) for IgG2. The presence of bisecting GlcNAc was not influenced by pregnancy or postpartum and was similar in cases (range min-max IgG1 13.7 to 14.7%, range min-max IgG2 13.2 to 14.3%) and controls (range min-max IgG1 13.7 to 14.4%, range min-max IgG2 13.0 to 14.0%). Moreover uni- and multivariate analyses did not reveal any effect of the previously mentioned covariates on the presence of bisecting GlcNAc.

The presence of the bisecting GlcNAc is related to IgG galactosylation. The levels of galactosylation of IgG1 or IgG2 with bisecting GlcNAc were at a significant lower level than IgG1 or IgG2 without bisecting GlcNAc (range min-max IgG1+bisecting GlcNAc 38.2 to 44.8%, range min-max IgG2+bisecting GlcNAc 31.8 to 38.9%) at every timepoint (*P *< 0.0001), but showed a similar pattern in time.

### Ethics committee approval

All participants gave informed consent. The study is in compliance with the Helsinki Declaration and approved by the Ethics Review Board at the Erasmus MC University Medical Center Rotterdam, The Netherlands.

## Discussion

This study demonstrates the association between changes in IgG galactosylation and RA-disease activity during pregnancy and postpartum. The most prominent increase in galactosylation was observed in RA-patients that spontaneously improved during pregnancy, whereas the reverse was observed for the flare postpartum. Finally, a good correlation between IgG N-glycan galactosylation and sialylation was demonstrated.

IgG galactosylation and RA in relation to pregnancy have been studied previously. Our results are in line with a previous study in which galactosylation levels in RA are described during pregnancy using the lectin analysis method [1]. The application of the MALDI-TOF-MS allowed us to analyze IgG1 and IgG2 separately and to analyze specifically the Fc-fragment glycosylation (and galactosylation). This was in contrast to the lectin method which cannot distinguish between IgGs and determines a combined value for the Fc and Fab fragment glycosylation. Compared to previous literature, we studied a larger cohort with a longer follow-up time postpartum. This enabled the description of the postpartum flares and identification of factors that influence galactosylation. Moreover a control group was added. Based upon studies in one patient it has been suggested that pregnancy-induced remission is associated with a fixed galactosylation level [2]. In contrast we demonstrated that the level of clinical remission was associated with a different level of galactosylation per timepoint during pregnancy and postpartum.

We have shown that the IgG galactosylation changes take place simultaneously with the changes in RA disease activity. Therefore one could argue that changes in IgG galactosylation are a mere epiphenomenon accompanying changes in disease activity. However, the strongest argument that galactosylation of IgG, and in particular agalactosyl IgG itself, indeed plays a pathogenic role is derived from animal studies. In these studies arthritis could only be transferred by infusion of agalactosyl IgG [15].

The pro-inflammatory role of agalactosyl IgG may be explained in multiple ways: first, IgG can act as an auto-antigen itself in RA. Since RF preferentially binds to agalactosyl IgG, this would result in more pronounced RF-agalactosyl IgG interaction and hence more inflammation [16,17]. Secondly, the pathogenicity of agalactosyl IgG is thought to be associated with its ability to activate the complement pathway via binding to mannose-binding lectin (MBL) [18]. This hypothesis has been questioned recently based upon studies in MBL-deficient mice [3]. As a result of the absence of galactose, agalactosyl IgG antibodies also lack terminal sialic acid residues. These terminal sialic acid residues have recently been implicated in suppressing inflammation via the induction of inhibitory FcãRIIb expression in mice [19,20]. Our analyses revealed a good correlation between IgG N-glycan galactosylation and sialylation. However, IgG sialylation levels were low and did not exceed 10%. Whether the effect of galactosylation is mediated in humans through the presence of increased sialylation of IgG still needs to be elucidated. Nevertheless, sialylation seems to be an additional important modification of IgG during pregnancy.

Since we have shown that changes in IgG galactosylation levels are associated with improvement of RA during pregnancy and the flare postpartum, identification of factors that influence galactosylation might give insight into pathogenic mechanisms underlying RA and might be a lead for the development of future therapies. For this purpose multivariate analyses were performed. These revealed that mainly disease activity and timepoint in pregnancy remained as an explanatory parameter for galactosylation. However, in the multivariate analysis also use of prednisone (for IgG2) and sulfasalazine (for IgG1) were associated with decreased galactosylation of IgG. Since decreased IgG galactosylation has been shown to be associated with more severe disease activity it is unlikely that this association is a direct consequence of the mode of action of these effective medications for RA. Although speculative, it is more likely that this association is related to the fact that both medications are only used during pregnancy by patients with a more aggressive RA and hence serve as markers for those patients with more severe RA.

Pregnancy-induced changes in cytokine or hormonal levels could be a possible explanation for the changes in galactosylation during pregnancy and postpartum. It has been suggested that IL-6 [21] or pregnancy-associated hormones like estrogen [17] or prolactin [22] could induce altered glycosyltransferase (or other (iso)enzyme) activity in B-cells that could result in immunoglobulins with different glycoforms.

For the first time it has been shown that the levels of bisecting GlcNAc are not influenced by pregnancy. Pekelharing *et al *found no changes in the presence of GlcNAc during pregnancy using gas-liquid chromatography [8], not distinguishing between antenna GlcNAc and the bisecting GlcNAc on other positions. The clinical relevance of bisecting GlcNAc is still unknown. Interestingly, the levels of IgG galactosylation with bisecting GlcNAc were significantly lower than the levels of IgG without bisecting GlcNAc.

## Conclusions

This large prospective cohort study demonstrates the association between IgG galactosylation changes with pregnancy-induced improvement and postpartum flare in RA-patients. Since IgG galactosylation was associated with sialylation, also sialylation seems to be an additional important modification during pregnancy. The levels of IgG galactosylation largely depend on the trimester of pregnancy or the timepoint of visit postpartum and disease activity, even after correction for medication use.

Future studies should focus on unraveling the exact mechanism behind the changes in IgG galactosylation and sialylation and on the consequences of these changes on the function of IgG itself during pregnancy and postpartum.

## Abbreviations

AcN: acetonitrile; ACR: American College of Rheumatology; CCP: cyclic citrullinated peptide; CRP: C-reactive protein; DAS28: Disease activity score 28; EULAR: European League Against Rheumatism; Fab: Fragment antigen-binding; Fc: Fragment crystalizable; Gal: galactose; GlcNAc: N-Acetylglucosamine; IgG: immunoglobulin G; IgG-G0: agalactosyl IgG, Gal-0, no galactose; IgG-G1: Gal-1, galactose on one arm; IgG-G2: Gal-2, galactose on both arms; MALDI-TOF-MS: matrix-assisted laser desorption/ionization - time of flight - mass spectrometer; MBL: mannose-binding lectin; Mon: months; MTP: microtitre plate; PARA-study: Pregnancy-induced Amelioration of Rheumatoid Arthritis-study; PP: postpartum; RA: rheumatoid arthritis; SA: sialic acid; SAS: Statistical Analysis Software; SD: standard deviation; SPE: solid phase extraction; SPSS: statistical package for the social sciences; TFA: trifluoroacetic acid; Trim: trimester of pregnancy; Wk: weeks.

## Competing interests

The authors declare that they have no competing interests.

## Authors' contributions

FG and RD had full access to all of the data in the study and take responsibility for the integrity of the data and the accuracy of the data analysis. FG, MW, MS, AD, MH and RD designed the study. FG, MW, MS and YM were involved in acquisition of the data. FG, MW, MS, SW, MH and RD analyzed the data of the MALDI-TOF-MS and interpreted the data. The manuscript was prepared by FG, MW, SW, MS, YM, AD, MH and RD. FG and SW did the statistical analyses. All authors read and approved the final manuscript.
